# Application of artificial intelligence in diagnosing COVID-19 disease symptoms on chest X-rays: A systematic review

**DOI:** 10.7150/ijms.76515

**Published:** 2022-09-28

**Authors:** Jakub Kufel, Katarzyna Bargieł, Maciej Koźlik, Łukasz Czogalik, Piotr Dudek, Aleksander Jaworski, Maciej Cebula, Katarzyna Gruszczyńska

**Affiliations:** 1 Department of Biophysics, Faculty of Medical Sciences in Zabrze, Medical University of Silesia, Jordana 19, 41-808 Zabrze, Poland; 2 Faculty of Medical Sciences in Katowice, Medical University of Silesia, 40-752 Katowice, Poland; 3 Division of Cardiology and Structural Heart Disease, Medical University of Silesia, 40-635 Katowice, Poland; 4 Professor Zbigniew Religa Student Scientific Association at the Department of Biophysics, Faculty of Medical Sciences in Zabrze, Medical University of Silesia, Jordana 19, 41-808 Zabrze, Poland; 5 Department of Radiology and Nuclear Medicine, Faculty of Medical Sciences in Katowice, Medical University of Silesia, 40-754 Katowice, Poland

**Keywords:** artificial intelligence, COVID-19, chest X-rays, convolutional neural network, diagnostic imaging

## Abstract

This systematic review focuses on using artificial intelligence (AI) to detect COVID-19 infection with the help of X-ray images.

**Methodology**: In January 2022, the authors searched PubMed, Embase and Scopus using specific medical subject headings terms and filters. All articles were independently reviewed by two reviewers. All conflicts resulting from a misunderstanding were resolved by a third independent researcher. After assessing abstracts and article usefulness, eliminating repetitions and applying inclusion and exclusion criteria, six studies were found to be qualified for this study.

**Results**: The findings from individual studies differed due to the various approaches of the authors. Sensitivity was 72.59%-100%, specificity was 79%-99.9%, precision was 74.74%-98.7%, accuracy was 76.18%-99.81%, and the area under the curve was 95.24%-97.7%.

**Conclusion**: AI computational models used to assess chest X-rays in the process of diagnosing COVID-19 should achieve sufficiently high sensitivity and specificity. Their results and performance should be repeatable to make them dependable for clinicians. Moreover, these additional diagnostic tools should be more affordable and faster than the currently available procedures. The performance and calculations of AI-based systems should take clinical data into account.

## Introduction

The ease with which the COVID-19 pandemic has spread highlights the necessity for the early detection of infection and the isolation of patients. Currently, the gold standard for diagnosing these patients is the reverse transcription-polymerase chain reaction (RT-PCR) test 1,2. However, due to a lack of availability of the tests in some areas, the possible false-negative results (caused by a low viral load), the high cost of testing and the delay in results delivery, the usage of diagnostic imaging tools, such as chest X-ray (CXR) and computed tomography (CT), plays an important role in treating patients 3,4,5. These diagnostic tools are used not only in the clinical diagnosis of patients with confirmed or presumed SARS-CoV-2 virus infection but also in assessing the risk of complications or possible progression and stating the stage of the disease. Diagnostic imaging helps differentiate COVID-19 infection from other pulmonary diseases 6,7,8. CT, due to its high sensitivity, is the imaging method of choice in diagnosing COVID-19 patients 3. CT assessment also seems promising in the clinical segregation of patients 9,10. Several significant correlations were found between chest CT examinations in people infected with SARS-CoV-2 virus and the number and percentage of lymphocytes, the percentage of neutrophils and the C-reactive protein and procalcitonin levels (p < 0.05 for all) 11. A relationship was also found between the severity of CT changes in patients hospitalised with COVID-19 pneumonia and the level of inflammatory cytokines, such as interleukin 6 and interleukin 2R 12,13. Therefore, it seems that chest CT in COVID-19 may be useful in prognosis assessment and in the prediction of the clinical outcomes of COVID-19.

Nevertheless, CXR has become more significant in the early detection of SARS-CoV-2 infection due to its wider access, capability of bedside examination, relatively low cost and shorter duration of the procedure 14-17. Several studies have investigated the relationship between the severity of changes observed in CXRs and the severity of the disease. A significant relationship was found between patients' need to be admitted to the intensive care unit and the probability of death in those who showed an increased extent and severity of lung opacity in the radiographic image 18. Relationships between the severity of COVID-19 and the need for hospitalisation, admission to the intensive care unit and the need for oxygen therapy were also demonstrated 19. In some patients, the radiological symptoms of SARS-CoV-2 infection occurred even before the serological confirmation of the disease, making it possible to introduce treatment before the appearance of clinical symptoms 20.

Recently, many studies on the detection of COVID-19 disease using CXR have been performed using various techniques with the help of artificial intelligence (AI) 21-23. Thus, the COVID-19 pandemic contributed to the rapid development of AI in radiology, which can be seen from the increase in scientific reports on this topic (after typing 'AI and radiology' in the public database PubMed, 1,105 articles (2018), 1,210 articles (2019), 1,666 articles (2020) and 1,916 articles (2021) were found). It was crucial to find new solutions that would be efficient enough to relieve the healthcare system 1. Then, many diverse information technology (IT) ideas were proposed, such as transfer learning techniques or novel network architectures, to improve CXR diagnostic performance in COVID-19 disease. In the initial period of the pandemic, an insufficient amount of image data limited the development of research on AI in COVID-19 diagnostics because there were no sufficiently large databases that could be used in scientific research 24,25. Currently, we have large, publicly available datasets that contain CXR from patients with active SARS-CoV-2 infection 26-28.

Several systematic reviews mentioning the use of CXR in COVID-19 diagnostics were conducted, but they did not exhaust the subject, treating it as an addition to the main part of the article that dealt with CT 29.

In a systematic review of the role of AI in the detection of COVID-19, Gudigar et al. examined CT, CXR and ultrasound of the lungs and found the disadvantages of CT, which had a 30-70 times higher radiation dose and lower availability than CXR. They also emphasised the high importance of lung ultrasound, which turned out to be an alternative to CXR, with comparable results presented by CT 30.

Alzubaidi et al. analysed 17 studies published from May 2020 to September 2020 on the use of deep learning (DL) technology to detect COVID-19 disease at an early stage using CXR, CT and ultrasound imaging. The authors showed that despite the significant effect of DL-based diagnostic methods on the early detection of COVID-19, many of them had not yet been tested in a clinical setting and required more research 31.

Due to the high number of similar articles, we conducted an extensive systematic review to systematise knowledge on the topic of CXR diagnostics in COVID-19. In the scope of this study, this paper provides a structured systematic review of the use of AI in interpreting the chest CXRs of patients with COVID-19. We present the objectives, methodology, technology and results from particular research articles included in this systematic review.

This systematic review analyses the various approaches of using AI in the diagnostic, therapeutic and treatment processes of COVID-19 infection with the help of patients' X-ray images.

## Methodology

To ensure the transparency and credibility of this study, this systematic review was conducted in accordance with the PRISMA 2020 Statement guidelines 32.

### Search strategy and selection criteria

**Figure 1:**
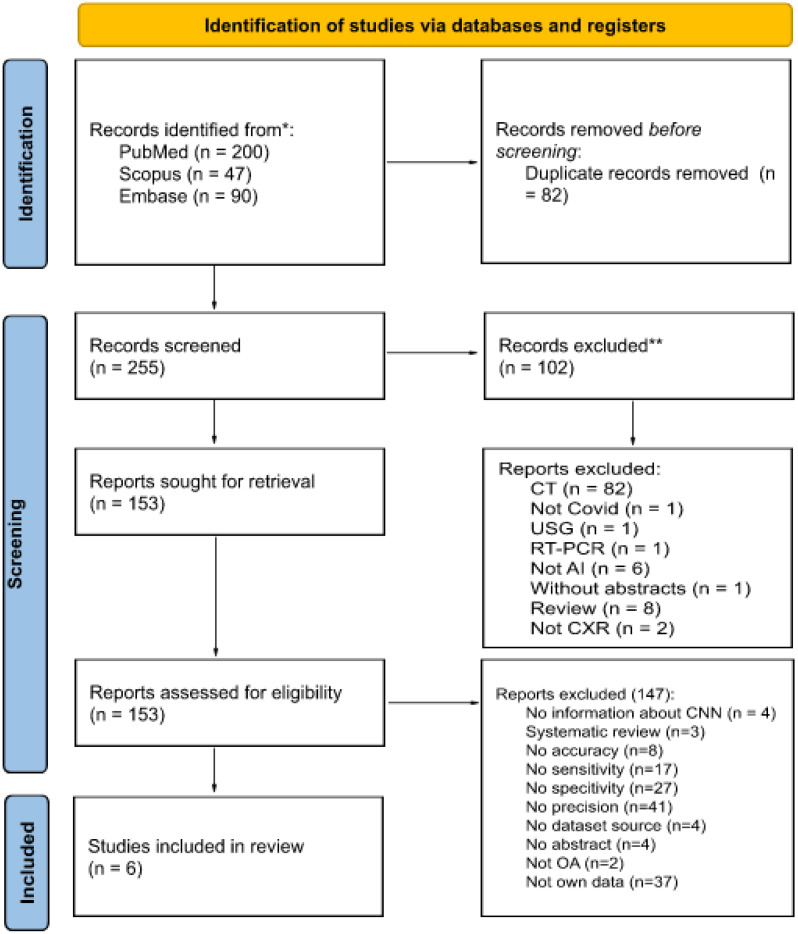
Number of articles in each systematic review process

The following databases were searched in January 2022: MEDLINE via PubMed, Embase and Scopus. The search was performed using the following medical subject heading terms: 'Artificial Intelligence', 'deep learning', 'machine learning', 'X-ray', 'chest' and 'COVID-19'. Searches were carried out by applying the following filters: articles published in the last five years, articles with abstracts, articles in English and articles concerning humans. Figure 1 shows the number of articles found and the number of articles excluded and included in this review, along with the detailed reasons.

### Data extraction and quality assessment

All found articles were imported into the Rayyan Qatar Computing Research Institute (QCRI) 33 and independently reviewed by two reviewers. The articles were evaluated for their usefulness in this study (i.e., research should contain information about using AI in the COVID-19 diagnostic process or in the detection of COVID-19 lung changes on X-ray images, should be reliable in terms of dataset size and should be about the use of CXR in diagnosing COVID-19 to some extent). Using the QCRI tool, 337 articles passed the first stage of the review, and 82 duplicates were excluded. A total of 102 articles that did not meet the inclusion criteria were excluded. Cohen's kappa was estimated to be 0.67 (agreement in 88.9%), which is interpreted as moderate substantial agreement 34. All conflicts (total number = 30) resulting from a misunderstanding were resolved by a third independent researcher after blinding the qualification results. Finally, 147 articles were excluded from the in-depth review of the articles' full text by the entire research team. In total, six studies met all the criteria and qualified for this study.

### Detailed inclusion criteria

The articles included in the study met all of the following requirements: articles written in English; articles with full-text available; articles with abstracts; articles about COVID-19, X-rays and AI; and articles with information about PPV, AUC or accuracy, sensitivity (recall), specificity and convolutional neural network (CNN); and articles with data used for CNN training, validating and testing that were collected in a research centre with which at least one of the authors was affiliated (own data).

### Detailed excluded criteria

Articles that were not included in this study met at least one of the following criteria: articles with no description of the CNN or other networks used in the research; systematic reviews or general reviews; articles with no information about AUC or accuracy in the results, sensitivity (recall), specificity, precision or datasets/dataset sources; articles on CT, USG or RT-PCR but not on X-rays; articles not concerning COVID-19; articles not concerning AI; and articles without abstracts or with full-text availability with payment (OA).

## Results

All six studies included in this systematic review were retrospective 35-40 and published in 2020 and 2021. Table 1 summarises the basic information about the studies. The authors used a total of 14,510 CXRs 35, 37-40. Only one article did not provide the number of CXRs used in the study 37. Half of the authors reported the number of patients (1,558 patients 35,36,38), while only two studies provided the number of both patients and the CXR 35,38. Due to the differences resulting from the different methods used to obtain CXR, the CXR number with (CXR with changes characteristic of COVID-19) or without a pathology could not be determined, except for one study that considered radiologically silent SARS-CoV-2 virus infection 38. It was also not possible to identify the exact number of photos per patient due to a lack of such information in all included articles. For example, 280 CXRs were obtained from 88 patients in one study 35, while 852 CXRs were obtained from 852 patients in another study 38.

### Methodology used in the articles

Chen et al. applied a four-step model based on the popular metaheuristic algorithm inspired by the Archimedes principle. The so-called Archimedes optimisation is used to adjust weights and train the network to minimise errors. It is an algorithm based on the population, with consideration of the immersed objects as candidates. Initially, the images are rescaled, normalised and then corrected using the histogram equation. Geometric, statistical and textural features are separated, while other less informative features are removed to improve the functioning of the system. The next and last stage is the classification of features based on an optimised multilayer perceptron network 35.

The diagnostic solution developed by Xia et al. combines the use of COVID-19 clinical features and COVID-19 recognition deep features from CXR images. The classifier used for this purpose was created based on Alexnet, a deep neural network in which shallow layers process structural features, such as edges, shapes and textural changes. Directed semantic information—the presence of changes and signs of disease advancement—is determined using the deep layers of the network. The clinical features, in the form of clinical vectors, create a separate layer that can be combined with recognised deep features. All elements of the deep neural network were implemented using Pytorch-based tools 36.

Sharifrazi et al. used a combination of a Sobel filter, support vector machine (SVM) and CNN. The application of data augmentation (width and height shifts, rotation and brightness changes) can improve the performance of the neural network. Before uploading the images into the CNN, they are passed through a Sobel filter to visualise the edges of the images. The 2D-CNN neural network used in this study is not a previously trained model. It consists of two main layers: convolutional layers (which are responsible for extracting features from the image) and fully connected layers (FC). The images then go to the SVM, which is a classifier with a 10-fold cross-validation strategy used in place of the sigmoid activation function (e.g. Softmax, Softplus, Leaky ReLU [LReLU] and Tanh) in the fully connected layers. In this way, the desired effect is achieved with a small amount of data 37.

Tabik et al. used a model called COVID-SDNet. In addition to the deep CNN based on the Resnet-50 architecture (with the last layer removed), this model contains a layer of 512 neurons with ReLU activation and a layer of 2-4 neurons with Softmax activation. All layers are tuned accordingly. Before sending the images to the network, they are qualitatively prepared through segmentation to eliminate redundant data. The next stage is the class-inherent transformation (CiT) network, which consists of FuCiTNet and the CiT method inspired by generative adversarial networks. The data are then classified using the CNN (Resnet-50 with ImageNet weights). An optimiser with a batch size of 16 and stochastic gradient descent was used 38.

Joshi et al. used DarkNet-53 as the main architecture in their research model and used secondary networks to improve feature extraction. DarkNet-53 has been pre-trained and consists of 53 layers, with 3 × 3 and 1 × 1 filters with shortcut connections. Training time is reduced, and efficiency improves with the use of pre-trained weights and transfer learning. DarkNet layers are applied to the core network layers, creating a combination of 106 network layers. This model allows for the detection of larger and smaller objects due to its multiscale, high-speed detection and high precision. Objects are detected on three different scales, and an advanced activation function is used: LReLU. To obtain sharp features (e.g. edges), max-pooling is used with convolutional layers 39.

A model with a proprietary architecture called CovXNet is proposed in Tanvir et al.'s research. The training process is divided into two parts using transfer learning. In the first phase (pretraining), a large dataset is used (1) containing photos of healthy patients and images of pneumonia not caused by COVID-19 disease. In the second phase (fine-tuning), a small dataset is used (2) containing pictures of pneumonia caused by COVID-19. It should be noted that some of the layers trained using a larger dataset (1) are frozen during the second phase. The CovXNet architecture mainly consists of a sequence of residual units, each consisting of several depth-wise convolutional layers with varying dilatation rates connected in parallel. The entire system is based on several CovXNet models optimised for different dimensions of the input photos connected in parallel with the final element: the meta learner. The meta learner aggregates the results from the above-mentioned models to provide the final prediction. Apart from the prediction itself, a visualisation technique based on gradients (localisation algorithm) is used, which allows for the marking of areas that contribute to a positive result 40.

### Study aims

Chen et al. used a CXR analysis system to facilitate the diagnostic process. This was achieved by using a new algorithm for the final classification of photos into two groups: those that did not show changes characteristic of COVID-19 and those with visible pathology (present features of COVID-19). The system is based on network weights and introduces modifications to previously used algorithms to reduce the complexity of extraction features. These features are raw data that have proven useful for statistical analysis. In this study, the main parameters of extraction were the geometric features, statistical features and image texture information 35. Xia et al. proposed that the CXR classifier could quickly and safely make an accurate diagnosis. This was achieved with the help of a deep neural network (DNN). The features derived from the CXR were divided into two groups: shallow features (e.g. edges, shapes and textures) and deep features (e.g. disease stage). These two groups of features were treated as matrices of gray values ​​and as clinical results (fever, nasal mucosa congestion, sore throat, throat mucosa congestion and sputum). Finally, they were combined, and a batch normalisation layer was added. The obtained results contributed to the final diagnosis 36. Sharifrazi et al. modified their own CXR classifier in three ways to determine which one is the most effective and the advantages and disadvantages of these modifications. These modifications were the addition of a Sobel filter, CNN-SVM or CNN-sigmoid and the modifications of the last two mentioned with the Sobel filter. The authors used a proprietary designed network in their study 37.

Tabik et al. proposed several extensions that included four severity levels of lung lesions (normal, mild, moderate and severe). All images were taken from PCR-positive patients to increase the effectiveness of the diagnosis. To achieve this, the COVID Smart Data-based Network (COVID-SDNet) method was proposed, which links the segmentation and modification of data to the appropriate CNN to draw conclusions, allowing for the identification of the disease 38. Joshi et al. proposed the creation of a mechanism that relies on DL to automate the process of correct COVID-19 identification. A binary classification system that gives better results than multi-class (three- or four-class) classification methods was introduced. The study consisted of collecting and analysing CXR data containing the features of haze opacity with lung consolidation. These images helped to train the CNN network model to ensure a high precision rate and fast lesion detection. The obtained experimental data were assessed in terms of various performance parameters (e.g. specificity, sensitivity, precision, F1-score and accuracy) 39. Tanvir et al. proposed AI systems used to detect COVID-19 and other pneumonias to make a rapid diagnosis with the help of DL.

### Datasets description

All of the works included in this paper were based on datasets created from data that were at least partially collected by the authors themselves. Using data collected at the hometown of at least one of the authors was one of the inclusion criteria. It should be noted that due to the difficulty in collecting self-collected data, their amount was relatively small (280 CXRs in 35 and 333 CXRs in 20) or constituted a small percentage of the data used in the study as a kind of supplement to publicly available datasets (11.61% in 39). In 39, self-collected data were used only in the final stage of network testing, as shown in Table 2.

The datasets of the studies included in this review differed from each other, not only in the number of collected CXRs but also in the categories into which the collected CXRs were divided. In half of the studies, the authors used a binary division of categories of the accumulated CXRs (COVID-19-positive (+) vs. healthy or bacterial pneumonia or NON-COVID-19 36-39, respectively), as shown in Table 3. However, the original division of CXR applied during the data collection stage was not necessarily the same as that used when creating the network 39,40.

The CXR photos included in the datasets were mostly from 2020 (January 2017-June 2020 in 19 and February-April 2020 in 20). The CXRs of healthy patients and those with non-covid pneumonia were obtained from earlier years to increase the amount of non-covid CXRs and to fine-tune the CNN to detect COVID-19 36.

Using data from one's own repository allows for relating imaging tests to clinical data. The studies in which the authors decided to collect and use clinical data focused on linking radiological symptoms to clinical symptoms and outcomes. This process improved the diagnostic efficiency of the network 36. However, many studies did not take clinical data into account 35,39,40, used sparse clinical data or did not use them at all in the process of creating a network, as they were only an additional element describing the dataset 37,38.

### CNNs used in the articles

In most of the articles included in this review, the authors created entirely original computational models based on various techniques that enhanced the desirable features of CXRs while discarding those considered less informative. All the models analysed the CXRs used clinically in diagnostic and therapeutic processes 35-40. The introduction of clinical features into the computational process (i.e. laboratory tests, symptoms, comorbidities, demographic features and outcome) contributed to a significant increase in the effectiveness of the analysis of patients' X-rays 36. Additional frameworks, tools and procedures were used to multiply the successive layers 37-39 and filters 37-40 aimed at detailing the specific features of CXRs (Pytorch 19, vector machine 37, 2.5% pixel added on each side of CXR 38, DarkNet with 53 layers 39). This enabled us to focus more precisely on the selected features and correct the data analysis by increasing the efficiency of classification. Most of the authors used the fully connected layer 36-39 and the kernel filter 37-40. Some authors used the sigmoidal activation function (Softmax 20,23, ReLU 38-40). The authors used transfer learning methods 36,38,39, while others created ensemble models based on many neural networks, each of which, appropriately modified, was responsible for a different phase of the calculations 38-40. Some researchers classified COVID-19 cases using machine learning techniques 35,37,38,40 instead of DL methods 36-39, which contributed to the features extraction from the images and the achievement of high recognition scores. A summary of the neural networks used in the articles is presented in Table 4.

### Results

In this review, we considered works that provided sensitivity, specificity, precision and accuracy or AUC. One study did not mention accuracy 36, and three did not provide an AUC 35,38,39. The majority of the 147 excluded papers did not have the precision parameter in their results. All the works included in this review used a binary system and assigned the analysed photos into two groups. Half of the studies divided the patients into groups with COVID-19 disease and those without any disease 35,37,40. Two studies divided patients into those who had COVID-19 and those who had any disease other than COVID-19 or were healthy 38,39. In one study, the division concerned patients with COVID-19 and those with influenza pneumonia 36. The results were radically different, as were the calculation methods used by the authors. Sensitivity was the first parameter considered in this review. The scores ranged from 72.59% to 100%. The relatively highest sensitivity was achieved by studies that divided patients into those with COVID-19 and those without any other disease (96%-100%) 35,37,40. In terms of the specificity parameter, the results obtained by the authors ranged from 79% to 99.9%. For the precision parameter, the obtained results were in the range of 74.74%-98.7%. In one study, the authors also included the values ​​of the precision parameter in the calculations for excluding (not confirming) COVID-19 disease in patients 38. For the accuracy parameter, the range was 76.18%-99.81%, and the relatively highest values ​​were achieved by studies dividing the patients into those with COVID-19 and those without any other disease (96%-100%) 35,37,40. One study did not include accuracy in its results 36. The AUC parameter was in the range of 95.24%-97.7%, but some authors did not report these values ​​in their results 35,38,39. Table 5 summarises the results.

## Discussion

The number of studies on the use of AI in diagnosing COVID-19 has grown exponentially since 2020, and the quality of the articles varied 41.

Some of the artificial neural network models described in this review showed high performance. This suggests that the implementation of such solutions and their integration with existing IT systems could help radiologists in their work, increasing their efficiency and effectiveness. The process of preparing and processing photos for analysis by some authors and the designed computational models differed, showing different approaches and diverse possibilities for CXR analysis.

The authors of one study conducted a validation study by comparing the diagnostic accuracy of the tested model to the results achieved by experienced doctors. Regardless of access to clinical data, AI not only performed significantly better (the AUC achieved by AI with and without clinical information was 0.935 and 0.958, respectively, vs. that achieved by pulmonary physicians with and without clinical information was 0.467 and 0.473, respectively), but the diagnostic process was also much faster (0.2 s vs. 25 min) 36.

Murphy et al. assessed the ability of an AI system (CAD4COVID-X-ray; Thirona) to classify chest radiographs and compared its performance with the descriptions of six radiologists. The study used a kit containing 454 chest radiographs of patients with suspected COVID-19 pneumonia. The AI ​​correctly classified CXR as COVID-19 pneumonia, with an AUC of 0.81, surpassing each of the radiologists at their highest possible sensitivity 42.

However, according to a systematic review by Roberts et al. 43, none of the studies included in their review were suitable for regular use in clinical practice due to the presence of numerous errors in the collected datasets or the insufficient validation procedure. In particular, the authors of the review pointed out the use of premade and generally available datasets. They emphasised that these collections did not contain information about the positive RT-PCR test results confirming the infection with the SARS-CoV-2 virus. Attention was also paid to the examination projections (anterior-posterior and posterior-anterior), as computational models could misclassify the characteristic image of a certain projection as a more or less severe degree of disease and not as an actual radiographic result. The authors also indicated the need to consider demographic data as datasets before applying a given computational model; only two of the six studies described in this review used these data 36,37.

The number of publicly available datasets is growing along with an increase in research on the use of AI in COVID-19 diagnostics. Nevertheless, the overall number of high-quality studies is negligible, and a lack of prospective studies and external verification is the greatest disadvantage. Most authors obtained data from various collections or institutions to ensure an adequate number of CXRs showing COVID-19, other pathological conditions and pathology-free images 44,45, often based only on their own data to a certain extent. Some authors made the datasets they created publicly available 38.

Therefore, the authors of this review considered it justified to conduct a multi-centre prospective study based on a unified methodology that could eliminate the problem of a small number of CXRs with an appropriate amount of clinical-demographic data.

Other authors paid attention to the sampling process of large datasets to reduce predictive uncertainty, even though most authors used relatively small samples because of a lack of access to large, open COVID-19 datasets 46-49 or did not apply them at all due to the methodology used in the study 36,50. In a systematic review, Santosh et al. analysed articles from 2020 describing AI-based diagnostic imaging (CT and CXR) tools and their performance, depending on the complexity of these tools and the size of the dataset. However, the authors showed that the performance of both types of models (CT and CXR) did not improve, depending on the size of the dataset 51. The data used during the creation and training of new neural networks should be properly balanced. According to Wei et al., even small differences of 30%-40% in the minority class can significantly affect the results obtained by researchers 52. Only one of the articles described in this review used numerically equal classes 38. Some of the studies used a small number of CXRs, which was probably due to the use of data from a self-created repository and the difficulty in collecting such a large number of CXRs in the first months of the pandemic 35,37,53.

Some studies included in this review were characterised by a great variety of applied IT solutions, making it difficult to compare the results obtained by the authors to individual works. Of all the studies included in this review, Sharifrazi et al. showed the highest sensitivity of 100% 20. Tabik et al.'s study was the only one that scored below 90%, with 72.59% 21. Three works obtained specificity higher than 90% 37-40. Most of the studies achieved precision above 90% 35-37,39,40, and only one achieved 78.67% (true positive) and 74.74% (true negative) 38.

Some researchers classified COVID-19 cases using machine learning techniques rather than DL methods 36-39 by extracting features from images and achieving high recognition scores. The solutions proposed by the authors may contribute to the development of new computational models that can help develop the technical and medical sciences.

Despite the enthusiastic approach of researchers and the suggestions that CXR should be a first-line diagnostic method for the detection and screening of COVID-19 cases 54, organisations such as the American College of Radiology 55, the Society of Thoracic Radiology and the American Society of Emergency Radiology 56 do not recommend routine imaging, especially CT, as a first-line diagnostic test for the detection of SARS-CoV-2 infection.

## Strengths and limitations of the study

Many articles that could be included in this review did not qualify because their focus was on the diagnosis of SARS-CoV 2 infection using CT, which was not of interest. The restrictive inclusion and exclusion criteria and the fact that other reviews, including systematic ones, were not considered resulted in the small number of studies included in this work.

The articles qualified for this study were analysed and described in detail with the utmost care and with particular attention to the methodology and results. As only articles containing their own CXR dataset were included in the study, this may contribute to the creation of more extensive databases and the development of AI in radiology.

## Conclusion

The development of accurate and highly sensitive AI-based computational models for the clinical evaluation and follow-up of COVID-19 patients is critical. Achieving sufficiently high sensitivity and specificity will allow the use of a network as an auxiliary tool in the diagnostic process. Prospective studies should be carried out to verify the correct functioning of the models, and external verification should be applied to the produced software to improve its quality. In the future, the developed solutions and designed tools can be used for the diagnosis of other diseases related to chest organs after appropriate transformations.

In conclusion, an ideal AI system evaluating CXRs in COVID-19 patients needs to be solid and stable. The results it provides must be within an acceptable range. It should be reliable and repeatable; that is, it should present similar results in many trials. It should be less expensive than the currently available solutions, for example, RT-PCR. In addition, the results must be verifiable by radiologists and pulmonologists and combined with the patients' symptoms and clinical data. AI systems should also take into account patients' age, gender and accompanying diseases, the symptoms of which could resemble those present in the course of COVID-19.

## Figures and Tables

**Table 1:** Articles used in the research

No.	Author	Work Title	Country
35	Chen et al.	A new optimal diagnosis system for coronavirus (COVID-19) diagnosis based on the Archimedes optimisation algorithm on chest X-ray images	China/Iran
36	Xia et al.	A rapid screening classifier for diagnosing COVID-19	China
37	Sharifrazi et al.	Fusion of convolution neural network, support vector machine and Sobel filter for accurate detection of COVID-19 patients using X-ray images	Iran/Australia/Singapore/USA/India/Taiwan
38	Tabik et al.	COVIDGR dataset and COVID-SDNet methodology for predicting COVID-19 based on chest X-Ray images	Spain
39	Joshi et al.	A deep learning-based COVID-19 automatic diagnostic framework using chest X-ray images	India/Czech Republic/ Italy/ Switzerland/ Spain
40	Mahmud et al.	CovXNet: A multi-dilation convolutional neural network for automatic COVID-19 and other pneumonia detection from chest X-ray images with transferable multi-receptive feature optimisation	Bangladesh

**Table 2:** Number of CXRs and patients in the articles

Citation number	Number of CXRs/patients in the datasets	Self-collected CXRs (%)	Public data (%)
35	280/88	100%	-
36	-/618	100%	-
37	333/-	100%	-
38	852/852	100%	-
39	6884/-	11.61%	88.39%
40	6161/-	100%	-

**Table 3:** Primary division of the collected CXRs into categories

Article	COVID-19 category	NON-COVID			
		Healthy	Bacterial pneumonia	Viral pneumonia	Other anomalies
35	+	+	-	-	-
36	+	-	+	-	-
37	+	+	-	-	-
38	+	+			
39	+	+	+	+	+
40	+	+	+	+	-

**Table 4:** CNNs used in the articles

No.	CNN
35	Proposed proprietary approach (based on a four-step computer-aided design (CAD)-based COVID-19 X-ray diagnosis system)
36	Alexnet (used for CXR processing and combined with clinical vector-created DNN)
37	2D-CNN (CAD-based; fusion of convolutional neural network, support vector machine and Sobel filter)
38	COVID-SDNet
39	DarkNet-53
40	CovXNet

**Table 5:** Results of the calculation parameters

Number	Image classes included	Sensitivity (%)	Specificity (%)	Precision (%)	ACC	AUC
35	COVID/normal	96	79	96	86	-
36	COVID/influenza pneumonia	91.54	81.19	94.92	-	0.9524
37	COVID/normal	100	95.23	98.70	99.02	0.9770
38	COVID/non-COVID	72.59	79.76	78.67(P)/74.74(N) *	76.18	-
39	COVID/non-COVID	98.45	99.90	98.45	99.81	-
40	COVID/normal	97.8	94.7	96.3	97.4	0.969

*P means true positives, and N means true negatives.

## Abbreviations

AI: artificial intelligence
AUC: area under the curve
AUC ROC: area under the curve receiver operating characteristics
CAD: computer-aided design
CiT: class-inherent transformations
CNN: convolutional neural network
COVID-19: coronavirus disease 2019
COVID-SDNet: COVID smart data-based network
CT: computed tomography
CXR: chest X-ray
DF: deep features
DL: deep learning
DNN: deep neural network
FC: fully connected
GAN: generative adversarial networks
ICU: intensive care unit
LReLU: leaky rectified linear unit
MeSH: medical subject headings
MLP: multilayer perceptron
PPV: precision
QCRI: Qatar Computing Research Institute
RT-PCR: real-time polymerase chain reaction
SARS-CoV-2: severe acute respiratory syndrome coronavirus 2
SGD: stochastic gradient descent
SVM: support vector machine
US: ultrasound sonography
